# Diverging Maternal and Cord Antibody Functions From SARS-CoV-2 Infection and Vaccination in Pregnancy

**DOI:** 10.1093/infdis/jiad421

**Published:** 2023-10-10

**Authors:** Emily H Adhikari, Pei Lu, Ye Jin Kang, Ann R McDonald, Jessica E Pruszynski, Timothy A Bates, Savannah K McBride, Mila Trank-Greene, Fikadu G Tafesse, Lenette L Lu

**Affiliations:** Division of Maternal-Fetal Medicine, Department of Obstetrics and Gynecology, University of Texas Southwestern Medical Center, Dallas, Texas, USA; Parkland Health, Dallas Texas, USA; Division of Infectious Diseases and Geographic Medicine, Department of Internal Medicine, University of Texas Southwestern Medical Center, Dallas, Texas, USA; Division of Infectious Diseases and Geographic Medicine, Department of Internal Medicine, University of Texas Southwestern Medical Center, Dallas, Texas, USA; Division of Infectious Diseases and Geographic Medicine, Department of Internal Medicine, University of Texas Southwestern Medical Center, Dallas, Texas, USA; Division of Maternal-Fetal Medicine, Department of Obstetrics and Gynecology, University of Texas Southwestern Medical Center, Dallas, Texas, USA; Department of Microbiology and Immunology, Oregon Health and Science University, Portland, Oregon, USA; Department of Microbiology and Immunology, Oregon Health and Science University, Portland, Oregon, USA; Department of Microbiology and Immunology, Oregon Health and Science University, Portland, Oregon, USA; Department of Microbiology and Immunology, Oregon Health and Science University, Portland, Oregon, USA; Parkland Health, Dallas Texas, USA; Division of Infectious Diseases and Geographic Medicine, Department of Internal Medicine, University of Texas Southwestern Medical Center, Dallas, Texas, USA; Department of Immunology, University of Texas Southwestern Medical Center, Dallas, Texas, USA

**Keywords:** pregnancy, SARS-CoV-2, neutralization, antibody Fc effector functions, IgG glycosylation

## Abstract

Maternal immunity impacts the infant, but how is unclear. To understand the implications of the immune exposures of vaccination and infection in pregnancy for neonatal immunity, we evaluated antibody functions in paired peripheral maternal and cord blood. We compared those who in pregnancy received mRNA coronavirus disease 2019 (COVID-19) vaccine, were infected by severe acute respiratory syndrome coronavirus 2 (SARS-CoV-2), and the combination. We found that vaccination enriched a subset of neutralizing activities and Fc effector functions that was driven by IgG1 and was minimally impacted by antibody glycosylation in maternal blood. In paired cord blood, maternal vaccination also enhanced IgG1. However, Fc effector functions compared to neutralizing activities were preferentially transferred. Moreover, changes in IgG posttranslational glycosylation contributed more to cord than peripheral maternal blood antibody functional potency. These differences were enhanced with the combination of vaccination and infection as compared to either alone. Thus, Fc effector functions and antibody glycosylation highlight underexplored maternal opportunities to safeguard newborns.

## Teaser

To understand how the immune exposures of vaccination and infection in pregnancy impact the neonate, this study evaluated paired peripheral maternal and cord blood. The data show that antibody Fc effector functions and glycosylation are underexplored opportunities to safeguard newborns.

Newborns are vulnerable to infections because their immune systems are immature. Maternal immunization can be leveraged to protect the infant, but available vaccines are limited for the pregnant compared to general population. If we understand how maternal immunity generated in pregnancy is transferred to the fetus, then we can improve prenatal approaches to prevent infectious disease related morbidity and mortality in the newborn.

Compared to the general population, pregnant individuals and infants <6 months old have higher risk of acute complications from coronavirus disease 2019 (COVID-19) [1]. Long-term sequelae are still being recognized in adults; in infants emerging data suggest that neurodevelopment delay can occur with in utero exposure to severe acute respiratory syndrome coronavirus 2 (SARS-CoV-2) [2, 3]. mRNA immunization in pregnancy is associated with decreased risk of maternal and infant short-term complications [4, 5], providing an opportunity to dissect the mechanisms by which the immune exposures of virus from infection and viral proteins from vaccination influence outcomes of subsequent SARS-CoV-2 challenge.

Although a spectrum of immunity contributes to defense against SARS-CoV-2 [6, 7], the primary form transferred to the fetus is antibodies, specifically immunoglobulin G (IgG). COVID-19 mRNA vaccination and SARS-CoV-2 infection induce IgG that carry neutralizing activities and antibody Fc effector functions [6–11]. Neutralizing activities inhibit viral entry into the cell [11, 12]. Antibody Fc-Fc receptor (FcR) engagement induce immune cell effector functions [13, 14] that prevent disease and block viral spread [7, 11, 15–17]. Diversity of the Fc domain through differential subclass and posttranslational glycosylation modulates binding to FcRs and the spectrum of effector functions [8, 10, 13, 14, 18–20]. Animal studies show that neutralizing and Fc functions can synergize [7, 11, 15–17]. However, only a limited number of human studies have evaluated them in parallel to enable direct comparisons [8, 9]. Expanding beyond neutralization allows for the development of tools that can overcome limitations of immune protection primarily dependent on binding to inhibit a single antigen such as the receptor binding domain (RBD).

In the pregnant population, a breadth of neutralizing activities and Fc effector functions are carried by peripheral maternal IgG and transferred across the placenta into cord blood [21–25]. Transfer occurs primarily through the high affinity FcRN, although low affinity FcRs could also be involved [21, 26, 27]. Thus, differences between peripheral maternal and cord blood IgG subclass and glycosylation [21, 26–31] could reflect characteristics required for localization. Moreover, changes in features could reflect maternally derived antibody functions in the neonate that could be protective in subsequent pathogen challenge. For COVID-19, vaccination induces higher RBD IgG than infection [21–24, 32]. Whether these higher titers are skewed towards neutralizing activities or Fc functions, what antibody features drive these functions, and how functions and features are impacted by placental transport are less clear.

The overall objective of this study is to dissect maternal-fetal antibody neutralizing activities and Fc functions from vaccination and infection in pregnancy. The secondary objective is to identify the antibody features that mediate activities and functions. Paired maternal peripheral and cord blood samples were collected at delivery from individuals who received mRNA COVID-19 vaccine, were infected by SARS-CoV-2, or the combination during pregnancy. Neutralization against live SARS-CoV-2 and the RBD-specific Fc functions were evaluated. To assess how different antibody features drive functions, relative levels of RBD-specific antibody isotype, subclass, and posttranslational glycosylation were determined. The data show that compared to infection, vaccination enriched a subset of maternal neutralizing and RBD Fc functions that was driven by IgG1 and was minimally impacted by antibody glycosylation. In paired cord blood, immunization also enhanced IgG1 and neutralizing activities, but compared to peripheral maternal blood, cord antibodies had more Fc functions. Additionally, changes in RBD IgG sialylation and fucosylation had greater impact on antibody functional potency in cord than matched peripheral maternal blood, and this was accentuated in the combination of vaccine and infection than either alone. Thus, maternal and fetal antibodies after infection and vaccination in pregnancy partially diverge.

## METHODS

### Study Design and Approval

Pregnant individuals were approached in ambulatory and inpatient settings at Parkland Health. Eligibility was determined by age (≥18 years) and immune exposure in pregnancy evaluated by report of symptoms and review of electronic health records from Parkland and regional health centers (Table 1, Supplementary Table 1, and Supplementary Figure 1). This observational study was approved by institutional review boards from University of Texas Southwestern and Parkland Health (STU2020-0375, STU2020-0214), and Oregon Health and Science University (PROTO202000015). Written informed consent was received.

**Table 1.** Clinical Characteristics of Study Patients

	Infection	Vaccine	Vaccine and Infection	*P* Value
Characteristic	(n = 22)	(n = 19)	(n = 28)	
Age, y	28.3 ± 6.5	32.4 ± 5.8	34.4 ± 6.7	.005
Race/ethnicity				.151
Hispanic	20 (91)	17 (90)	28 (100)	
Black, non-Hispanic	2 (9)	1 (5)	0 (0)	
White, non-Hispanic	0 (0)	1 (5)	0 (0)	
Other				
Maternal BMI at first visit, kg/m^2^	32 (26–36)	30 (27–33)	34 (30–39)	.147
Male infant sex	12 (55)	12 (63)	15 (54)	.789
EGA at delivery, wk	38 (36–38)	38 (38–39)	37 (37–38)	.300

Data shown as No. (%), mean ± standard deviation (SD), or median (Q1–Q3) as appropriate.

Abbreviations: BMI, body mass index; EGA, estimated gestational age.

### Sample Collection

Paired peripheral maternal and cord blood were collected at or within 24 hours of deliveries by venipuncture or umbilical vein in acid citrate dextrose (ACD) and serum separator tubes (SST). Plasma and serum were isolated by centrifugation, aliquoted, stored at −80°C, and heat-inactivated prior to use.

### Focus Reduction Neutralization Test

SARS-CoV-2 clinical isolates were passaged once [8]: USA-WA1/2020 (original strain; BEI NR-52281); hCoV-19/USA/PHC658/2021 (B.1.617.2; BEI NR-55611), and hCoV-19/USA/CO-CDPHE-2102544747/2021 (B.1.1.529-BA.2; BEI NR-56520). Sera incubated with viral particles were added to Vero E6 cells. Wells were overlayed with 1% methylcellulose, fixed (4% formaldehyde), permeabilized (0.1% bovine serum albumin, 0.1% saponin), incubated with 1:5000 anti-SARS-CoV-2 serum from alpaca immunized with RBD and nucleocapsid (Capralogics, Inc), then 1:20 000 anti-alpaca-HRP (NB7242, Novus), and developed with TrueBlue (SeraCare). Foci were imaged with CTL Immunospot Analyzer, enumerated using viridot package, and percent neutralization calculated relative to the average of virus-only wells. Fifty percent focus reduction neutralization tests (FRNT_50_) were determined by fitting percent neutralization to a 3-parameter logistic model [8].

### Antigen-Specific Antibody Isotype, Subclass, and FcR Binding

Microspheres (Luminex) were coupled by 1-ethyl-3-[3-dimethylaminopropyl] carbodiimide hydrochloride and *N*-hydroxysulfosuccinimide (ThermoScientific) to RBD (BEI NR-52309), spike (BEI NR-52308), Delta spike (BEI NR-55614), Omicron spike (BEI NR-56447), and a mixture of influenza antigens (BEI NR-20083, NR-51702, NR-12148). Antigen-coupled microspheres were incubated with sera. Bead-bound antigen-specific antibodies were detected using phycoerythrin (PE)-coupled anti-IgG/anti-IgA1/anti-IgM/anti-IgG1/anti-IgG2/anti-IgG3/anti-IgG4 (1 µg/mL; Southern Biotech). For FcR binding, FcγRIIIa/CD16a, FcγRIIa/CD32a, FcγRIIb/CD32b, and FcRN (R&D Systems) labeled with PE (Abcam) were used (1 µg/mL). PE signals were measured on a MAGPIX (Luminex). Replicate samples over 3 serial dilutions were tested in 2 independent experiments. Representative data were chosen by the highest signal-to-noise ratio [8, 33].

### Antibody-Dependent Complement Deposition

Antigen-coated microspheres were incubated with heat-inactivated sera (37°C, 2 hours). Guinea pig complement (Cedarlane) 1:60 was added (37°C, 20 minutes), then anti-C3 PE-conjugated goat polyclonal IgG (1 µg/mL; MP Biomedicals). C3 deposition was quantified on MAGPIX (Luminex) as detailed above [8].

### Antibody-Dependent Cellular Phagocytosis

RBD (BEI NR-52309) was biotinylated with Sulfo-NHS-LC Biotin (ThermoFisher) and incubated with neutravidin beads (Invitrogen) (4°C, 16 hours). Antigen-coupled beads were incubated with sera (37°C, 2 hours) and then THP1 cells (37°C, 16 hours). Bead uptake was measured on a BD-LSR Fortessa and analyzed by FlowJo 10. Replicate samples over 3 serial dilutions were tested in 2 independent experiments. Representative data were chosen by the highest signal-to-noise ratio [8, 34].

### Antibody-Dependent Natural Killer Cell Activation

Sera were added to enzyme-linked immunosorbent assay (ELISA) plates coated with RBD (300 ng/well; BEI NR-52309) (37°C 2 hours). CD16a.NK-92 cells (PTA-6967, American Type Culture Collection) with brefeldin A (Biolegend), Golgi Stop (BD Biosciences), and anti-CD107a (clone H4A3, BD Biosciences) were then added (37°C, 5 hours). Cells were stained with anti-CD56 (clone 5.1H11, BD Biosciences) and anti-CD16 (clone 3G8, BD Biosciences) and fixed (4% paraformaldehyde [PFA]). Intracellular cytokine staining to detect interferon-γ (IFN-γ; clone B27, BD Biosciences) and tumor necrosis factor-α (TNF-α; clone Mab11, BD Biosciences) was performed in permeabilization buffer (Biolegend). Markers were measured by flow cytometry as detailed above [8].

### All and RBD-Specific IgG Glycosylation

RBD (BEI NR-52309) was biotinylated with sulfosuccinimidyl-6-[biotinamido]-6-hexanamido hexanoate (ThermoScientific) and coupled to streptavidin beads (New England Biolabs). Sera were incubated with RBD-coupled beads. RBD-specific antibodies were eluted using 100 mM citric acid (pH 3.0) and neutralized with 0.5 M potassium phosphate (pH 9.0). IgG was isolated by protein G (Millipore) and deglycosylated using PNGase (New England Biolabs). Glycans were labeled with 8-aminoinopyrene-13,6-trisulfonic acid (ThermoFisher), unbound removed using Agencourt CleanSEQ beads (Beckman Coulter) (all IgG glycans) and Bio-Gel P-2(Bio-rad) (RBD-specific glycans), quantitated using ABI 3500xL, and analyzed with GlycanAssure version 1.0 [8, 35, 36].

### Statistical Analyses

Statistical analyses were performed and graphs plotted using R 4.1.2, Stata 17, GraphPad 9.0, Excel 365, and Cytoscape 3.9.1. Data were evaluated for normality and independence of the residuals, heteroscedasticity and linear relationships between dependent and independent variables, and log transformed as needed to meet these assumptions. Linear regression models were used to adjust for maternal age and body mass index and determine the effects of fetal sex and disease severity. Wilcoxon-matched pair signed rank tests were used to compare maternal-cord pairs. Simple linear regression was used to examine the relationships between IgG subclass and antibody functions and IgG glycoforms and antibody functional potencies. For tables, analysis of variance was used for age as it was normally distributed, Kruskal-Wallis test was used for all other continuous variables and χ^2^ and Fisher exact tests for categorical variables. *P* values (2-sided) <.05 were considered significant.

## RESULTS

### Study Subjects

We collected matched peripheral maternal and cord blood at deliveries (17 February 2021 to 27 May 2022) from individuals who in pregnancy received mRNA vaccination and/or were SARS-CoV-2 infected at Parkland Health, Dallas County's public hospital in Texas. Pregnant individuals were categorized into 3 immune exposure groups (infection only, vaccine only, and the combination vaccine plus infection) by history, documentation of immunization and SARS-CoV-2 nasal swab polymerase chain reaction (PCR), and independent determination of nucleocapsid IgG (Table 1 and Supplementary Figure 1). Infections occurred across WA1, Delta, and Omicron periods, spanning asymptomatic to critical. Two vaccine doses were received by 68%, with the last dose in the third trimester (Supplementary Figure 1) and no differences between BNT162b2 and mRNA-1273 (Supplementary Table 2). The age of the infected-only group was lower than the vaccine plus infection group but body mass index (BMI), gestational age at delivery (75% full-term), infant sex, and 21 additional clinical outcomes were not different (Table 1 and Supplementary Table 1). With 94% Hispanic, the racial/ethnic distribution in this study represents a subset of the Parkland patient population (Supplementary Table 3) [37].

### SARS-CoV-2 Neutralizing Activities

**Figure 1.**
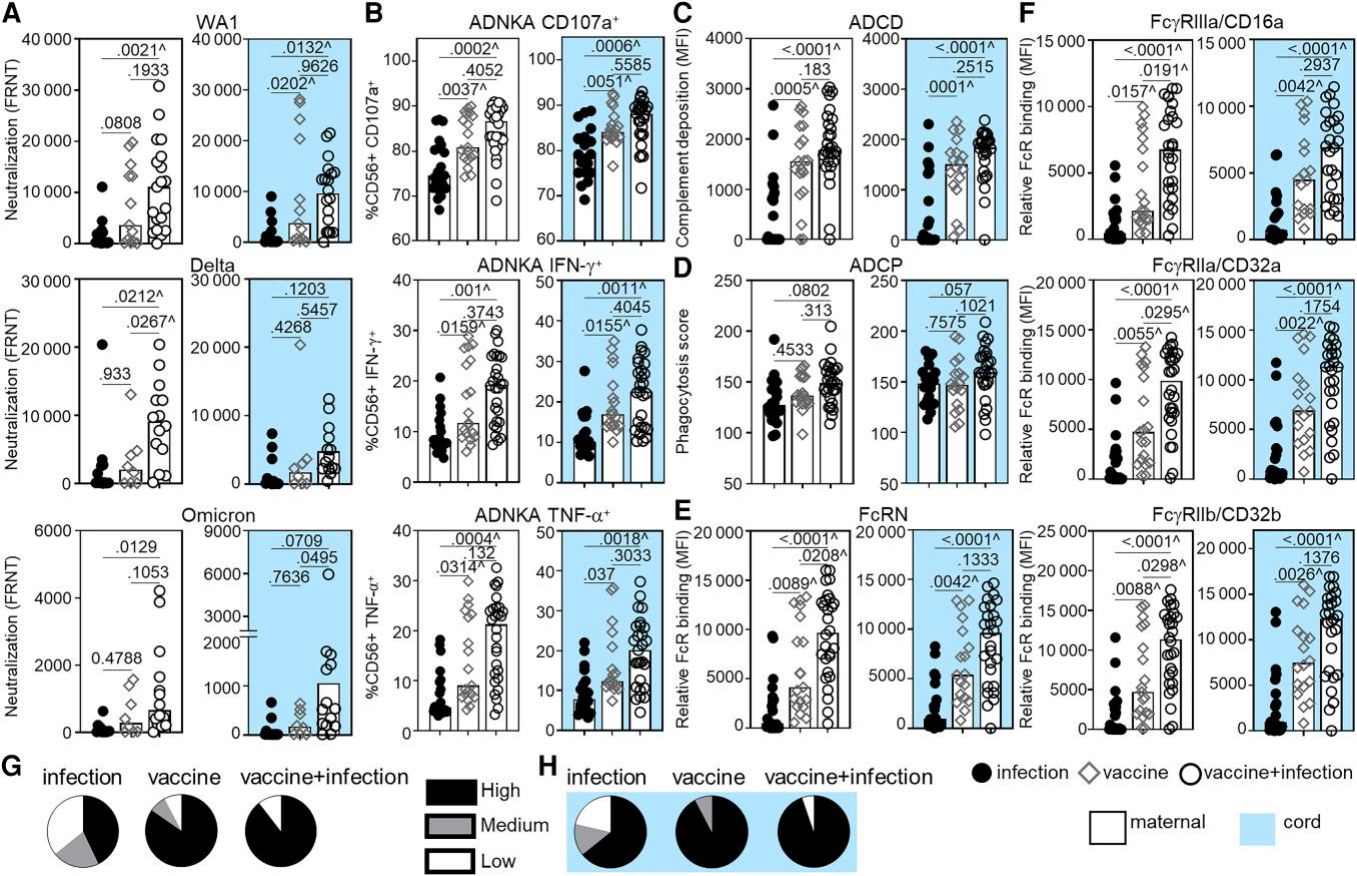
Increasing maternal and cord blood neutralizing and Fc effector functional magnitude and breadth were observed after infection, vaccination, and vaccination and infection in pregnancy. The bars depict the median of matched maternal (white, left) and cord (blue, right) blood levels of (*A*) neutralization (FRNT_50_) against SARS-CoV-2 WA1 (infection n = 14, vaccine n = 13, vaccine + infection n = 19), Delta, and Omicron viruses (infection n = 12, vaccine n = 8, vaccine + infection n = 14); (*B*) ADNKA by CD107a, IFN-γ, and TNF-α; (*C*) ADCD; (*D*) ADCP; and relative binding to (*E*) FcRN and (*F*) FcγRIIIa/CD16a, FcγRIIa/CD32a, and FcγRIIb/CD32b specific to receptor-binding domain. *B*–*F*, sample sizes are infection n = 20, vaccine n = 18, vaccine + infection n = 27. *P* values are adjusted for maternal age and body mass index using linear regression. ^ Marks significance after adjustment for multiple comparisons by Benjamini-Hochberg. The proportion of detectable functions was used to categorize individuals as a high, medium, or low responder (Supplementary Figure 4). Polyfunctional breadth is depicted as the percentages of each type of responder within each group in (*G*) maternal and matched (*H*) cord samples. Abbreviations: ADCD, antibody-dependent complement deposition; ADCP, antibody-dependent cellular phagocytosis; ADNKA, antibody-dependent natural killer cell activation; FRNT_50_, 50% reduction neutralization test; IFN-γ, interferon-γ; MFI, mean fluorescence intensity; SARS-CoV-2, severe acute respiratory syndrome coronavirus 2; TNF-α, tumor necrosis factor-α.

We first measured neutralizing activity [12]. Because 40% of infections in this study occurred when WA-1 was predominant, we performed FRNTs using the clinical SARS-CoV-2 strain (WA1/2020) (Supplementary Figure 2). We adjusted *P* values for maternal age and body mass index as potential confounders [32, 38, 39]. Maternal and cord neutralizing activities were lowest after infection, higher with vaccine, and highest with vaccine plus infection (Figure 1*A*). Because 28% and 26% of infections occurred during the Delta and Omicron periods, respectively, we next tested these variants and found similar increases (Figure 1*A*). Thus, independent of the initial variant of infection, immunization induced higher neutralizing activity against all variants.

### RBD Fc Effector Functions

Decay studies show that antibody Fc functions are more durable than neutralization [9] and data from animal models show that Fc-FcR engagement limits disease [7, 15–17]. We examined the RBD-specific Fc functions of antibody-dependent natural killer cell activation (ADNKA) which leads to cellular cytotoxicity, antibody-dependent complement deposition (ADCD), and antibody-dependent cellular phagocytosis (ADCP). The lowest levels of ADNKA (Figure 1*B*) and ADCD (Figure 1*C*) were after infection, higher with vaccine, and highest with vaccine plus infection, with significance after adjustment for multiple comparisons. However, ADCP was minimally impacted (Figure 1*D*). These data show that a subset of maternal and cord Fc functions marked vaccination.

Fc domain engagement of low-affinity FcRs initiates effector functions while the high-affinity FcRN mediates placental transport and recycling [14, 40]. Binding to FcRN (Figure 1*E*) and the low-affinity activating FcγRIIIa/CD16a and FcγRIIa/CD32a, and inhibitory FcγRIIb/CD32b (Figure 1*F*) were lowest after infection, higher with vaccine, and highest with vaccine plus infection. Thus, enhanced FcR binding was associated with vaccination.

Infant sex [41], prematurity [42], trimester [21, 22], vaccine platform [22], and disease severity [24] impact antibodies. Within the limited power of this study to test these variables, there were no significant associations (data not shown). Consistent with findings in the general population [43], there were no differences with respect to the order of vaccine and infection (Supplementary Figure 1).

Because SARS-CoV-2 spike protein, in particular RBD which binds to ACE2, is essential for viral entry into a host cell [12], this antigen is shared between the virus and mRNA vaccines [44, 45]. As such, RBD correlated with spike-specific IgG (Supplementary Figure 3*A* and 3*B*). Similarly, correlations between IgG (Supplementary Figure 3*A* and 3*B*) and FcγRIIIa/CD16a (Supplementary Figure 3*C* and 3*D*) reactive to the original and Delta and Omicron spike variants were significant. Consistent with findings in the general population and animal models [6, 7, 46], immune exposure to mRNA vaccine and SARS-CoV-2 generated antibodies capable of responding to a spectrum of variants (Figure 1*A* and Supplementary Figure 3).

Beyond the magnitude of a single antibody function, greater polyfunctional antibody breadth is associated with protection [8, 15–17]. For each individual sample, we categorized the proportion of detectable neutralizing and Fc functions as high (>90%), medium (80%–90%), or low (<80%) (Supplementary Figure 4). A larger proportion of high polyfunctional breadth was in the vaccinated than infected (Figure 1*G* and 1*H*).

### Maternal-Fetal Transfer of Antibody Functions

**Figure 2.**
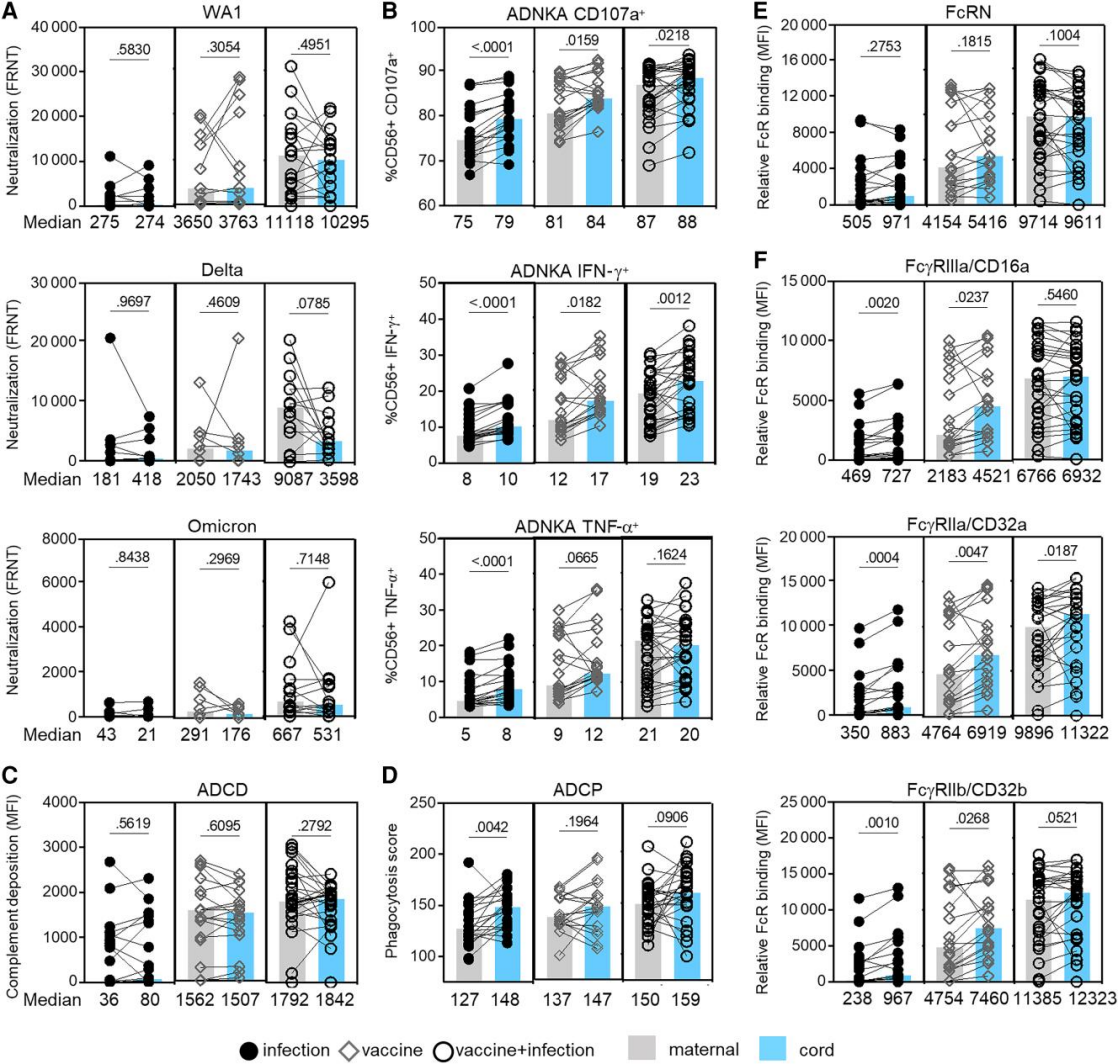
Fc effector functions are preferentially transferred across the placenta compared to neutralizing activities. The bars depict the median of matched maternal (grey) and cord (blue) blood levels of (*A*) neutralization against live SARS-CoV-2 WA1, Delta, and Omicron; (*B*) ADNKA; (*C*) ADCD; (*D*) ADCP; and relative binding to (*E*) FcRN and (*F*) FcγRIIIa/CD16a, FcγRIIa/CD32a, and FcγRIIb/CD32b specific to receptor-binding domain. Statistical significance was calculated by Wilcoxon-matched pairs test. Abbreviations: ADCD, antibody-dependent complement deposition; ADCP, antibody-dependent cellular phagocytosis; ADNKA, antibody-dependent natural killer cell activation; FRNT, reduction neutralization test; IFN-γ, interferon-γ; MFI, mean fluorescence intensity; SARS-CoV-2, severe acute respiratory syndrome coronavirus 2; TNF-α, tumor necrosis factor-α.

To understand the effect of immune exposure on placental transfer, we compared peripheral maternal and cord blood antibody activities and functions. Neutralizing activities did not differ (Figure 2*A*). In contrast, ADNKA was lower in maternal than cord blood (Figure 2*B*). This was not observed for ADCD (Figure 2*C*). For ADCP, this occurred after infection and less after vaccine and vaccine plus infection (Figure 2*D*). No difference was observed for the high-affinity FcRN that mediates IgG placental transport (Figure 2*E*). Consistent with the low-affinity FcγRs modulating ADNKA and ADCP, relative binding for FcγRIIIa/CD16a, FcγRIIa/CD32a, and FcγRIIb/CD32b were lower in maternal than cord blood (Figure 2*F*). This difference occurred in the infection group and vaccine group, and not the combination. Together, these data show that in contrast to neutralization and ADCD, there was preferential transport to the fetus of Fcγ receptor functions that can be influenced by immune exposure.

### RBD-Specific Isotype, Subclass, and Antibody Functions

**Figure 3.**
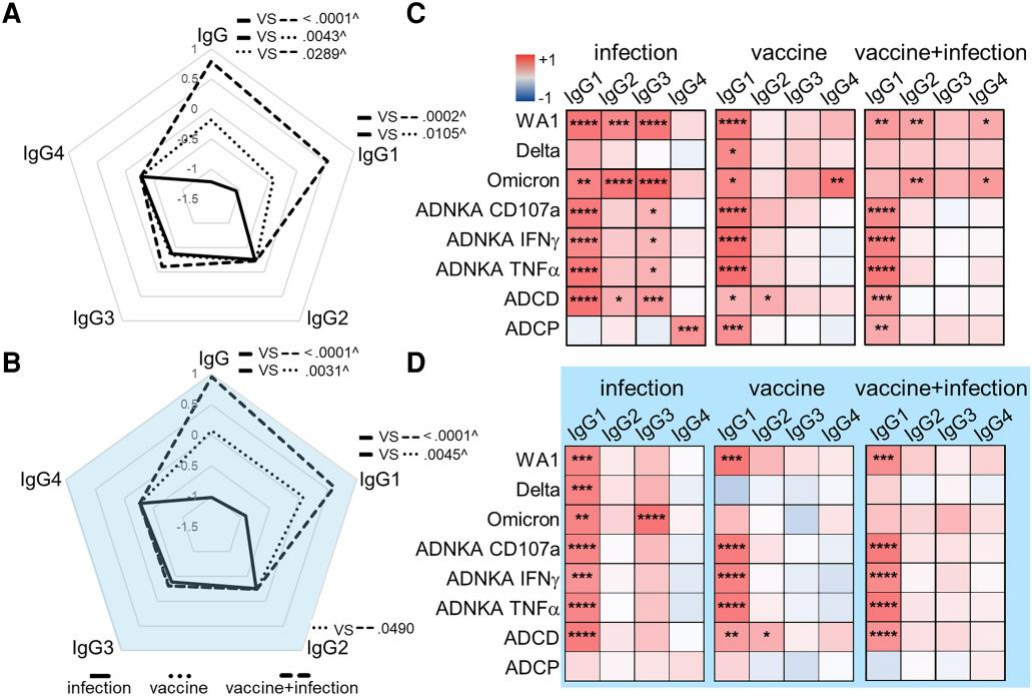
IgG1 is the primary subclass that drives antibody functions. Radar plots summarize the magnitude of RBD-specific IgG and subclass in (*A*) maternal and (*B*) matched cord blood. Each line represents the median Z-scored data for each group (infection n = 20, vaccine n = 18, vaccine + infection n = 27). *P* values are adjusted for maternal age and body mass index using linear regression. ^ Marks significance after adjustment for multiple comparisons by Benjamini-Hochberg. Heatmaps of the regression coefficients (*r*^2^) summarize the dependency of RBD-specific antibody functions on subclasses in (*C*) maternal and (*D*) cord blood by simple linear regression. *P ≤ .05, **P ≤ .01, ***P ≤ .001, ****P ≤ .0001. Abbreviations: IFN-γ, interferon-γ; IgG, immunoglobulin G; RBD, receptor-binding domain; TNF-α, tumor necrosis factor-α.

To understand how antibody features drive functional diversity, we measured isotypes and subclasses [13]. In peripheral maternal blood, the magnitude of RBD and not control influenza IgG was enhanced with vaccination (Figure 3*A*, Supplementary Figures 5 and 6*A*). Specifically, RBD IgG1 was increased in maternal and cord blood (Figure 3*A* and 3*B*, and Supplementary Figure 6*B*), remaining significant after adjustment for multiple comparisons and contrasting IgM and IgA1 (Supplementary Figure 7).

Using linear regression, we assessed the dependency of neutralizing and Fc functions on RBD-specific subclasses. We found that IgG1 was the main subclass that drove antibody functions across immune exposures. However, IgG2–4 in addition to IgG1 linked to peripheral maternal blood functions (Figure 3*C*), contrasting cord blood functions that focused more solely on IgG1 particularly after vaccination (Figure 3*D*).

### RBD-Specific IgG Glycosylation and Antibody Functional Potency

**Figure 4.**
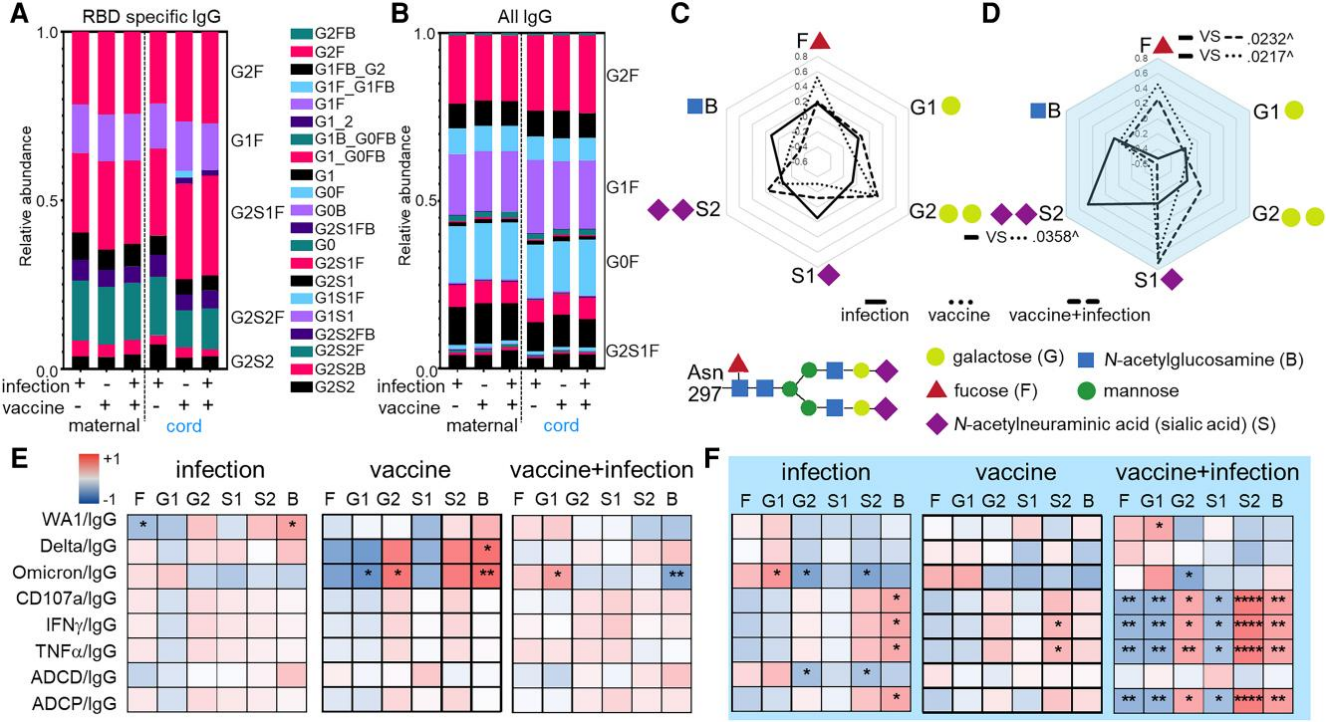
Antibody glycosylation impacts functional potency. The relative abundance of (*A*) RBD-specific and (*B*) all IgG individual glycoforms are depicted. Radar plots summarize (*C*) maternal and (*D*) cord blood glycoforms from RBD relative to all IgG for each sample with lines showing the median Z-scored data for each group. *P* values are adjusted for maternal age and body mass index using linear regression. ^ Marks significance after adjustment for multiple comparisons by Benjamini-Hochberg. Heatmap of the regression coefficients (*r*^2^) summarizes the dependency of RBD-specific antibody function potency on RBD-specific IgG glycans in (*E*) maternal and (*F*) cord samples by simple linear regression. Fucosylated (F), monogalactosylated (G1), digalactosylated (G2), monosialylated (S1), disialylated (S2), and bisecting *N*-acetyl-glucosamine (B) glycoforms are shown. *P ≤ .05, **P ≤ .01, ***P ≤ .001, ****P ≤ .0001. Abbreviations: IFN-γ, interferon-γ; IgG, immunoglobulin G; RBD, receptor-binding domain; TNF-α, tumor necrosis factor-α.

Differential posttranslational IgG glycosylation modulates Fc functions [8, 18, 19] and potentially placental transfer [21, 26, 27, 29]. A core biantennary structure of mannose and *N*-acetylglucosamine (GlcNAc) on a conserved N297 of the Fc domain is modified with the addition and subtraction of galactose, sialic acid, fucose, and bisecting GlcNAc into diverse forms [13, 47] (Figure 4 and Supplementary Figure 8). We determined the relative abundance of the individual glycoforms and found that on a global level, glycans on RBD-specific IgG were different from all IgG (Figure 4*A* and 4*B*). We calculated the abundance of all individual glycoforms containing fucose, sialic acid, galactose, and bisecting GlcNAc (Supplementary Figure 8*C*), quantitating the glycans on RBD-specific relative to all IgG. There were no changes observed on maternal IgG with respect to immune exposures (Figure 4*C* and Supplementary Figure 9*A*). However, compared to infection, vaccine groups had increased fucosylation and decreased disialylation on cord IgG (Figure 4*D* and Supplementary Figure 9*B*), which remained significant after adjustment for multiple comparisons. This contrasted the minimal changes in galactose and GlcNAc (Figure 4*C* and 4*D*, and Supplementary Figure 9). Thus, RBD-specific IgG glycosylation highlighted differences between paired peripheral maternal and cord blood after vaccination compared to infection.

Antibody glycosylation impacts functional potency [47]. Using linear regression, we found that functional potency in cord compared to maternal blood was more dependent on glycans (Figure 4*E* and 4*F*). The most prominent difference was observed in the vaccine plus infection group, highlighting the negative effect of fucose and positive of disialic acid, the only RBD IgG glycans that changed with immune exposures in cord blood (Figure 4*D*).

### Evaluating the Effects of Vaccination in Pregnancy on Maternal and Cord Antibodies

Regardless of whether maternal infection occurred, the intervention of mRNA vaccination in pregnancy is associated with decreased infant risk of SARS-CoV-2 morbidity and mortality [5]. To understand how the antibody findings in this study could inform on immunity associated with the intervention of immunization, we grouped all vaccinated pregnant individuals together (vaccine with and without [±] infection) to compare to the unvaccinated (infection only).

**Figure 5.**
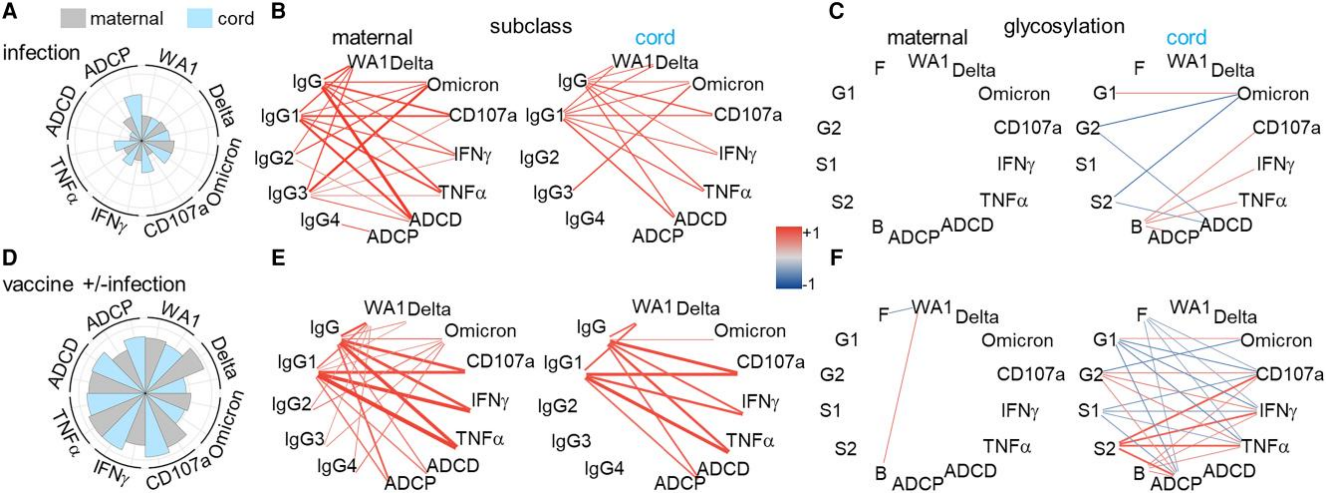
Subclass and glycosylation differentially drive neutralizing activities and Fc effector functions in peripheral maternal and cord blood in (*A*–*C*) the absence and (*D*–*F*) presence of vaccination in pregnancy. *A* and *D*, The magnitudes of maternal and cord blood functions are summarized in polar plots. Each slice represents the median Z-scored data for each group. Network matrices summarize the dependency of antibody functions on (*B* and *E*) IgG subclasses and (*C* and *F*) potency on glycans in maternal (left) and cord (right) blood by simple linear regression. Line colors represent regression coefficients, and width statistical significance (−log *P*). *P ≤ .05, **P ≤ .01, ***P ≤ .001, ****P ≤ .0001. Abbreviations: ADCD, antibody-dependent complement deposition; ADCP, antibody-dependent cellular phagocytosis; B, bisecting; F, fucosylated; G1, monogalactosylated; G2, digalactosylated; IFN-γ, interferon-γ; IgG, immunoglobulin G; S1, monosialylated; S2, disialylated; TNF-α, tumor necrosis factor-α.

In the absence of vaccination, neutralizing activities generated from prior SARS-CoV-2 infection predominated peripheral maternal blood whereas Fc functions highlighted by ADCP characterized cord blood (Figure 5*A*). Neutralizing activities and Fc functions depended on IgG1–4 in maternal while this was limited in cord blood to IgG1 and IgG3 (Figure 5*B*). There were no significant relationships between maternal IgG glycans and functional potencies, contrasting cord blood (Figure 5*C*). Thus, immune exposure to SARS-CoV-2 in pregnancy induced peripheral maternal immunity characterized by neutralizing activities and a breadth of IgG subclasses, diverging from the Fc functions driven by glycans in cord blood.

In the presence of vaccination, there were high peripheral maternal neutralizing activities and Fc functions (Figure 5*D*). The predominance of Fc functions in cord blood was less apparent in the presence compared to absence of vaccination (Figure 5*A* and 5*D*). The relationships between IgG1 and functions were all strengthened (Figure 5*E*). Moreover, the impacts of IgG glycans on functional potency expanded, particularly in cord blood (Figure 5*F*). Thus, vaccination in pregnancy induced an overall more balanced spectrum of maternal and fetal neutralizing and Fc functions. Immunization accentuated divergence between peripheral maternal and cord blood with IgG subclasses driving functions in the former and glycans latter.

## DISCUSSION

The data here show that infection, mRNA vaccination, and the combination of infection and vaccination in pregnancy generated parallel and diverging antibody activities, functions, and features in the maternal-fetal dyad. Infection induced the lowest, vaccination higher, and the combination of vaccination and infection the highest magnitude of peripheral maternal and cord antibody neutralizing activities and Fc functions (Figure 1 and Figure 5). Antibody-mediated viral neutralization, natural killer cell activation and cytotoxicity, and C1q-mediated complement activation were enhanced whereas monocyte phagocytosis was minimally altered (Figures 1 and Figure 5). IgG, specifically IgG1, was the primary antibody that increased in vaccination compared to infection (Figure 3*A* and 3*B*). As such, neutralizing activities and Fc functions became more dependent on IgG1 as opposed to other subclasses (Figure 3*C* and 3*D*, and Figure 5*E*). Notably, antibody functions in the vaccine and vaccine plus infection groups were more alike than the infection group (Figure 1 and Figure 3). Thus, the most important factor influencing the magnitude and polyfunctional breadth of RBD IgG1 driven antibody activities and functions where maternal and cord immunity parallel was vaccination.

Differences between peripheral maternal and cord blood occur with respect to (1) the impact of glycosylation on antibody functional potency, and (2) the balance between neutralizing activities and Fc functions. Vaccination compared to infection induced changes in cord and not matched peripheral maternal IgG glycosylation (Figure 4*A*–*D*, and Figure 5*C*). These involved fucose and disialic acid, which are associated with shifts in Fcγ effector functional potency such as ADNKA (Figure 4*F* and Figure 5*F*). Fc functions were also more prominent than neutralizing activities in cord than peripheral maternal blood (Figure 5*A*). Consistent with these observations, there was preferential transport across the placenta of Fc functions than neutralizing activities (Figure 2).

This study focuses on RBD-specific antibodies to directly compare the immune exposures of infection and vaccine. Because RBD is critical for host cell entry, neutralizing activities dominate how RBD-specific antibodies influence outcomes. However, SARS-CoV-2 infection generates antibodies that react to spike, matrix, and nucleocapsid with nonneutralizing Fc functions [11, 17, 21]. As such, these data likely underestimate the influence of virally induced Fc functions.

Other studies have reported differences between maternal and cord IgG glycans [21, 26, 29, 30]. None compare the same antigen-specific antibody after the different immune exposures of infection and vaccine that potentially impact clinical outcomes. Multiple glycosyltransferases and glycosidases in B cells mediate glycosylation [47]. The divergent impact of vaccination on peripheral maternal and cord blood antibody glycosylation suggests that differential placental transfer occurs potentially through low-affinity FcγRs [21, 26, 30]. Alternatively, B-cell extrinsic glycosylation enzymes could modify immune complexes as they traffic from the pregnant individual to the fetus [47].

The impacts of differences in maternal and fetal antibody glycosylation could be broad. All polyclonal IgG have *N*-linked glycans on the Fc domain that drive FcR binding and effector functions [13, 14, 47]; 20% have modifications on the Fab domain that influence stability, half-life, and avidity to antigens [35, 47]. Thus, antibody glycans can affect neutralizing and Fc functions, representing a source of diversity in the fetus where immunity passed from the mother is primarily IgG. Recent data in a mouse model of *Listeria* infection in pregnancy show that changes in IgG Fab domain sialylation modulates protection for pups through B-cell activation [31]. Thus, maternally derived IgG glycans can both directly and indirectly impact pathogens that challenge the neonate. Using vaccine adjuvants and platforms to elicit different glycan profiles [48] could provide new ways to enhance immunity for newborns.

Apart from influenza, no vaccine is licensed for use in pregnancy to specifically protect infants from disease. However, this strategy is used for SARS-CoV-2, tetanus, and pertussis. More recently, data show that administration of a bivalent prefusion F vaccine in pregnancy prevents respiratory syncytial virus (RSV)-associated illness in newborns [49] but the mechanisms are less clear. Neutralizing antibody activities were detected in the pregnant individual and transferred to infants but also induced by other vaccines with more limited efficacy [50]. Thus, neutralizing activities may not be linked to clinical outcomes but how Fc functions compare is not known. In cytomegalovirus (CMV) infection, protection from congenital transmission is associated with Fc functions [25]. The SARS-CoV-2 and mRNA vaccine data in this study show that this observation can extend beyond the pathogen CMV and immune exposure of infection. COVID-19 mRNA and RSV F vaccine overlapping signatures could provide principles to guide the design of more effective tools against CMV, group B *Streptococcus*, Zika, and other emerging pathogens disproportionately impacting the maternal-fetal dyad.

## Supplementary Data

Supplementary materials are available at *The Journal of Infectious Diseases* online. Consisting of data provided by the authors to benefit the reader, the posted materials are not copyedited and are the sole responsibility of the authors, so questions or comments should be addressed to the corresponding author.

## Supplementary Material

jiad421_Supplementary_Data

## Notes

***Author contributions***. E. A. obtained patient samples, collected clinical data, and prepared and revised the manuscript. P. L. designed and conducted experiments, analyzed data, and prepared and revised the manuscript. Y. J. K. assisted in conducting experiments, analyzing data, and preparing the manuscript. A. M. and J. P. analyzed the data. T. A. B., M. G. T., and S. K. M. conducted the neutralization assays and analyzed data. F. G. T. supervised neutralization assays, analyzed data, and provided critical revisions to the manuscript. L. L. L. conceived, designed, coordinated, and supervised the work, analyzed the data, and wrote and revised the manuscript.

***Acknowledgments***. We thank the patients, Brenda Espino, and the University of Texas Southwestern COVID-19 Biorepository (Dwight Towler, David Greenberg, Benjamin Greenberg, and Nancy Monson) for samples. We thank Catherine Spong and Trish Perl for their support. Gabrielle Lessen and Joshua Miles provided graphical assistance. Gabriella Kalantar assisted in manuscript review.

***Financial support***. This work was supported by University of Texas Southwestern (UTSW) Internal Medicine Pilot Project Award (to E. H. A. and L. L. L.); University of Texas Southwestern (UTSW) Disease Oriented Clinical Scholars Award (to L. L. L.); Burroughs-Wellcome Fund UTSW Training Resident Doctors as Innovators in Science (to Y. J. K.); National Institutes of Health (grant numbers T32HL083808 to T. A. B. and R01AI141549-01A1 to F. G. T.); Oregon Health and Science University Innovative IDEA grant (grant number 1018784 to F. G. T.); University of Texas Southwestern FOCUS program funded by the American Heart Association (to E. H. A.); Doris Duke Charitable Foundation (to E. H. A.); the Harry S. Moss Heart Trust (to E. H. A.); and Parkland Community Health Plan, a component unit of Dallas County Hospital District doing business as Parkland Health.
