# Supplementary material for: Diverging Maternal and Cord Antibody Functions From SARS-CoV-2 Infection and Vaccination in Pregnancy
Source: J Infect Dis. 2023 Oct 10;229(2):462–72. doi: 10.1093/infdis/jiad421 (PMC10873180; doi:10.1093/infdis/jiad421)
Supplement: jiad421_Supplementary_Data [file jiad421_supplementary_data.zip › 20230911_Supplemental table 1.docx]

Supplementary Table 1. Additional clinical characteristics of study patients

|  | **Infection** | **Vaccine** | **Vaccine and infection** | **P value** |
| --- | --- | --- | --- | --- |
| **Characteristic** | **n = 22** | **n = 19** | **n = 28** |  |
| Nulliparous | 9 (41) | 5 (26) | 3 (11) | 0.048I |
| Pregestational diabetes | 3 (14) | 2 (11) | 8 (29) | 0.266 |
| Chronic hypertension | 3 (14) | 5 (26) | 4 (14) | 0.562 |
| Preeclampsia with severe features | 3 (14) | 2 (11) | 7 (25) | 0.444 |
| Chorioamnionitis | 2 (9) | 0 (0) | 1 (4) | 0.481 |
| Prelabor rupture of membranes | 2 (9) | 0 (0) | 5 (18) | 0.139 |
| Induction of labor | 13 (59) | 8 (42) | 12 (43) | 0.439 |
| Cesarean delivery | 7 (32) | 11 (58) | 13 (46) | 0.241 |
| EGA <37 weeks at delivery | 7 (32) | 4 (21) | 6 (21) | 0.684 |
| Infant birth weight <10^th^ percentile | 2 (9) | 3 (16) | 5 (18) | 0.690 |
| Neonatal intensive care unit admission | 4 (18) | 0 (0) | 5 (18) | 0.130 |

Data shown as n (%), mean ± standard deviation (SD), or median (Q1-Q3) as appropriate.

BMI= body mass index, EGA= estimated gestational age, NICU= neonatal intensive care unit
