# Supplementary material for: Diverging Maternal and Cord Antibody Functions From SARS-CoV-2 Infection and Vaccination in Pregnancy
Source: J Infect Dis. 2023 Oct 10;229(2):462–72. doi: 10.1093/infdis/jiad421 (PMC10873180; doi:10.1093/infdis/jiad421)
Supplement: jiad421_Supplementary_Data [file jiad421_supplementary_data.zip › 20230911_Supplemental table 2.docx]

Supplementary Table 2: Comparison of clinical characteristics of individuals receiving mRNA-1273 and BNT162b2

|  | **mRNA-1273** | **BNT162b2** |
| --- | --- | --- |
| **Characteristic** | **n = 4** | **n = 43** |
| Age, years | 36.8 (4.7) | 33.3 (6.4) |
| Race/ethnicity |  |  |
| Hispanic | 4 (100) | 41 (95) |
| Black, non-Hispanic | 0 (0) | 1 (2) |
| White, non-Hispanic | 0 (0) | 1 (2) |
| Other | 0 (0) | 0 (0) |
| Nulliparous | 0 (0) | 8 (19) |
| BMI at first visit, kg/m^2^ | 37.5 (34.5-39.6) | 32.1 (28.5-36.4) |
| SARS-CoV-2 in pregnancy | 3 (75) | 25 (58) |
| mRNA Vaccination in pregnancy booster | 2 (50) | 7 (16) |
| Pregestational diabetes | 2 (50) | 8 (19) |
| Chronic hypertension | 1 (25) | 8 (19) |
| Preeclampsia with severe features | 2 (50) | 7 (16) |
| Chorioamnionitis | 0 (0) | 1 (2) |
| Induction of labor | 1 (25) | 19 (44) |
| Cesarean | 3 (75) | 21 (49) |
| EGA at delivery, weeks | 29.9 (27.5-33.2) | 25.4 (16.8-29.8) |
| EGA<37 weeks delivery | 2 (50) | 8 (19) |
| Infant birth weight <10^th^ percentile | 2 (50) | 6 (14) |
| NICU admission | 1 (25) | 4 (9) |
| Male infant sex | 1 (25) | 26 (60) |

Data shown as n (%), mean ± standard deviation (SD), or median (Q1-Q3) as appropriate.

BMI= body mass index, EGA= estimated gestational age, NICU= neonatal intensive care unit
