# Supplementary material for: Diverging Maternal and Cord Antibody Functions From SARS-CoV-2 Infection and Vaccination in Pregnancy
Source: J Infect Dis. 2023 Oct 10;229(2):462–72. doi: 10.1093/infdis/jiad421 (PMC10873180; doi:10.1093/infdis/jiad421)
Supplement: jiad421_Supplementary_Data [file jiad421_supplementary_data.zip › 20230911_Supplemental table 3.docx]

Supplementary Table 3. Characteristics of study cohort compared with deliveries at Parkland Health, 2021

|  | **Study patients** | **Deliveries 2021** |
| --- | --- | --- |
| **Characteristic** | **n = 69** | **n = 11,170** |
| Age, years | 31.9 ± 6.8 | 27.5 ± 6.4 |
| Race/ethnicity |  |  |
| Hispanic | 65 (94) | 8643 (77) |
| Black, non-Hispanic | 3 (4) | 1799 (16) |
| White, non-Hispanic | 1 (1) | 448 (4) |
| Other |  | 280 (3) |
| Nulliparous | 17 (25) | 3431 (31) |
| BMI at first visit, kg/m^2^ | 32 (28-37) | 29 (25-33) |
| Documented SARS-CoV-2 in pregnancy | 50 (72) | 2681 (24) |
| Any mRNA vaccination in pregnancy | 47 (68) | 2772 (25) |
| Pregestational diabetes | 13 (19) | 210 (2) |
| Chronic hypertension | 12 (17) | 847 (8) |
| Preeclampsia with severe features | 12 (17) | 1158 (10) |
| Chorioamnionitis | 3 (4) | 911 (8) |
| Prelabor rupture of membranes | 7 (10) | 2444 (22) |
| Induction of labor | 33 (48) | 3047 (27) |
| Cesarean delivery | 31 (45) | 3248 (29) |
| EGA at delivery, weeks | 38 (37-38) | 39 (38-40) |
| EGA <37 weeks at delivery | 17 (25) | 1057 (9) |
| Infant birth weight <10^th^ percentile | 10 (14) | 1173 (11) |
| NICU admission | 9 (13) | 572 (5) |
| Male infant sex | 39 (57) | 5774 (52) |

Data shown as n (%), mean ± standard deviation (SD), or median (Q1-Q3) as appropriate.

BMI= body mass index, EGA= estimated gestational age, NICU= neonatal intensive care unit
