# Supplementary material for: Diverging Maternal and Cord Antibody Functions From SARS-CoV-2 Infection and Vaccination in Pregnancy
Source: J Infect Dis. 2023 Oct 10;229(2):462–72. doi: 10.1093/infdis/jiad421 (PMC10873180; doi:10.1093/infdis/jiad421)
Supplement: jiad421_Supplementary_Data [file jiad421_supplementary_data.zip › 20230913_Supplemental figure 1 legends.docx]

**Supplementary Figure Legends**

**Supplementary Figure 1:** History, review of electronic medical record for documentation of vaccination and SARS-CoV-2 nasal PCR, and independent evaluation of SARS-CoV-2 nucleocapsid IgG were used to define infection and vaccine in pregnancy. Heatmap depicts results from SARS-CoV-2 nasal PCR (from the primary and regional medical clinics and hospitals), clinical disease severity with respect to COVID-19, SARS-CoV-2 nucleocapsid IgG in maternal and cord blood and documentation of BNT162b2 or mRNA-1273 vaccination during pregnancy. COVID-19 disease severity in pregnancy was classified as asymptomatic (no symptoms), mild (upper respiratory or mild febrile illness without lower respiratory symptoms), moderate (lower respiratory symptoms without oxygen requirement, with SpO2 ≥94% on room air), severe (oxygen requirement or SpO2 <94% on room air), or critical (respiratory failure, mechanical ventilation, or extracorporeal membrane oxygenation). Gestational age was determined by best obstetric criteria from last menstrual period and first ultrasound available in pregnancy. Pie charts depict the percentage of individuals who received vaccine doses (left) and when the last vaccine dose in pregnancy was administered (right).
