# Supplementary material for: Diverging Maternal and Cord Antibody Functions From SARS-CoV-2 Infection and Vaccination in Pregnancy
Source: J Infect Dis. 2023 Oct 10;229(2):462–72. doi: 10.1093/infdis/jiad421 (PMC10873180; doi:10.1093/infdis/jiad421)
Supplement: jiad421_Supplementary_Data [file jiad421_supplementary_data.zip › 20230913_Supplemental figure 2 legends.docx]

**Supplementary Figure Legends**

**Supplementary Figure 2:** FRNT50 were calculated from neutralization graphs generated in focus forming assays for each clinical SARS-CoV-2 virus (WA1, Delta, Omicron). Each graph shows the data for one individual sample. FRNT50 were determined by fitting %neutralization to a 3-parameter logistic model. Limit of detection (LOD) was defined by the lowest detection tested; values below were set to LOD-1. Duplicate FRNT50 values were calculated separately to confirm values were within 4-fold. When true, a final FRNT50 was calculated by fitting to combined replicates.
