# Supplementary material for: Diverging Maternal and Cord Antibody Functions From SARS-CoV-2 Infection and Vaccination in Pregnancy
Source: J Infect Dis. 2023 Oct 10;229(2):462–72. doi: 10.1093/infdis/jiad421 (PMC10873180; doi:10.1093/infdis/jiad421)
Supplement: jiad421_Supplementary_Data [file jiad421_supplementary_data.zip › 20230913_Supplemental figure 3 legends.docx]

**Supplementary Figure Legends**

**Supplementary Figure 3:** RBD, Spike and variant specific IgG and FcγRIIIa/CD16a binding correlate. Relationships between SARS-CoV-2 Wuhan-Hu1 RBD, Spike, and Delta B.1.617.2 and Omicron B.1.1.529 variant specific (A and B) IgG and (C and D) FcγRIIIa/CD16a in (A and C) maternal and (B and D) cord blood were assessed by Spearman correlation.
