# Supplementary material for: Diverging Maternal and Cord Antibody Functions From SARS-CoV-2 Infection and Vaccination in Pregnancy
Source: J Infect Dis. 2023 Oct 10;229(2):462–72. doi: 10.1093/infdis/jiad421 (PMC10873180; doi:10.1093/infdis/jiad421)
Supplement: jiad421_Supplementary_Data [file jiad421_supplementary_data.zip › 20230913_Supplemental figure 4 legends.docx]

**Supplementary Figure Legends**

**Supplementary Figure 4:** SARS-CoV-2 reactive polyfunctional breadth was calculated for each individual sample with all 12 features listed. Responses fell into three main categories: those with high (90-100%), medium (80-90%) and low (<80%) proportion of functions detected.
