# Supplementary material for: Diverging Maternal and Cord Antibody Functions From SARS-CoV-2 Infection and Vaccination in Pregnancy
Source: J Infect Dis. 2023 Oct 10;229(2):462–72. doi: 10.1093/infdis/jiad421 (PMC10873180; doi:10.1093/infdis/jiad421)
Supplement: jiad421_Supplementary_Data [file jiad421_supplementary_data.zip › 20230913_Supplemental figure 5 legends.docx]

**Supplementary Figure Legends**

**Supplementary Figure 5:** No changes are observed in control influenza hemagglutinnin (Flu) specific IgG levels in maternal blood. P values are adjusted for maternal age and body mass index using linear regression.
