# Supplementary material for: Diverging Maternal and Cord Antibody Functions From SARS-CoV-2 Infection and Vaccination in Pregnancy
Source: J Infect Dis. 2023 Oct 10;229(2):462–72. doi: 10.1093/infdis/jiad421 (PMC10873180; doi:10.1093/infdis/jiad421)
Supplement: jiad421_Supplementary_Data [file jiad421_supplementary_data.zip › 20230913_Supplemental figure 6 legends.docx]

**Supplementary Figure Legends**

**Supplementary Figure 6:** Increasing maternal and cord blood IgG and subclasses titers are observed after infection, vaccination, and the combination in pregnancy. Bars depict the median of RBD-specific (A) total IgG and (B) subclasses in maternal (white) and cord (blue) blood. P-values are adjusted for maternal age and body mass index using linear regression. ^ marks significant p values after adjustment for multiple comparisons by Benjamini-Hochberg.
