# Supplementary material for: Diverging Maternal and Cord Antibody Functions From SARS-CoV-2 Infection and Vaccination in Pregnancy
Source: J Infect Dis. 2023 Oct 10;229(2):462–72. doi: 10.1093/infdis/jiad421 (PMC10873180; doi:10.1093/infdis/jiad421)
Supplement: jiad421_Supplementary_Data [file jiad421_supplementary_data.zip › 20230913_Supplemental figure 7 legends.docx]

**Supplementary Figure Legends**

**Supplementary Figure 7:** RBD IgM and IgA1 in maternal blood do not differ with respect to immune exposure and are minimally detected in cord blood. (A) Dot plots show the magnitude of RBD IgM (left) and IgA1 (right) in maternal blood. P values are adjusted for maternal age and body mass index using linear regression. (B) The bars depict the median of matched maternal (grey) and cord (blue) levels of RBD IgM (left) and IgA1 (right). Statistical significance was calculated by Wilcoxon-matched pairs test.
