# Supplementary material for: Diverging Maternal and Cord Antibody Functions From SARS-CoV-2 Infection and Vaccination in Pregnancy
Source: J Infect Dis. 2023 Oct 10;229(2):462–72. doi: 10.1093/infdis/jiad421 (PMC10873180; doi:10.1093/infdis/jiad421)
Supplement: jiad421_Supplementary_Data [file jiad421_supplementary_data.zip › 20230913_Supplemental figure 8 legends.docx]

**Supplementary Figure Legends**

**Supplementary Figure 8:** Representative capillary electrophoresis chromatographs for one pair of maternal and infant cord samples show differences in glycoforms captured from RBD-specific and all IgG. Peaks are identified from (A) all and (B) RBD-specific IgG glycans captured and quantitated by relative abundance. (C) The collective relative abundance of all individual glycoforms with fucose (F), sialic acid (S), galactose (G) and bisecting n-acetyl-glucosamine (B) are calculated.
