# Supplementary material for: Diverging Maternal and Cord Antibody Functions From SARS-CoV-2 Infection and Vaccination in Pregnancy
Source: J Infect Dis. 2023 Oct 10;229(2):462–72. doi: 10.1093/infdis/jiad421 (PMC10873180; doi:10.1093/infdis/jiad421)
Supplement: jiad421_Supplementary_Data [file jiad421_supplementary_data.zip › 20230913_Supplemental figure 9 legends.docx]

**Supplementary Figure Legends**

**Supplementary Figure 9:** RBD-specific IgG glycosylation differs between infection and vaccination in cord and not paired maternal blood. RBD-specific relative to all IgG glycoforms containing fucose (F), monogalactosylated (G1), digalactosylated (G2), monosialylated (S1), disialylated (S2) and bisecting n-acetyl-glucosamine (B) structures are Z-scored to enable comparisons. Each individual dot represents a single sample and are depicted for (A) maternal and (B) cord blood (infection n=18, vaccine n=18, vaccine+infection n=19). Bars represent the median for each group. P values are adjusted for maternal age and BMI using linear regression. ^ marks significant p values after adjustment for multiple comparisons by Benjamini-Hochberg.
