# Supplementary figures and images for: Diverging Maternal and Cord Antibody Functions From SARS-CoV-2 Infection and Vaccination in Pregnancy
Source: J Infect Dis. 2023 Oct 10;229(2):462–72. doi: 10.1093/infdis/jiad421 (PMC10873180; doi:10.1093/infdis/jiad421)

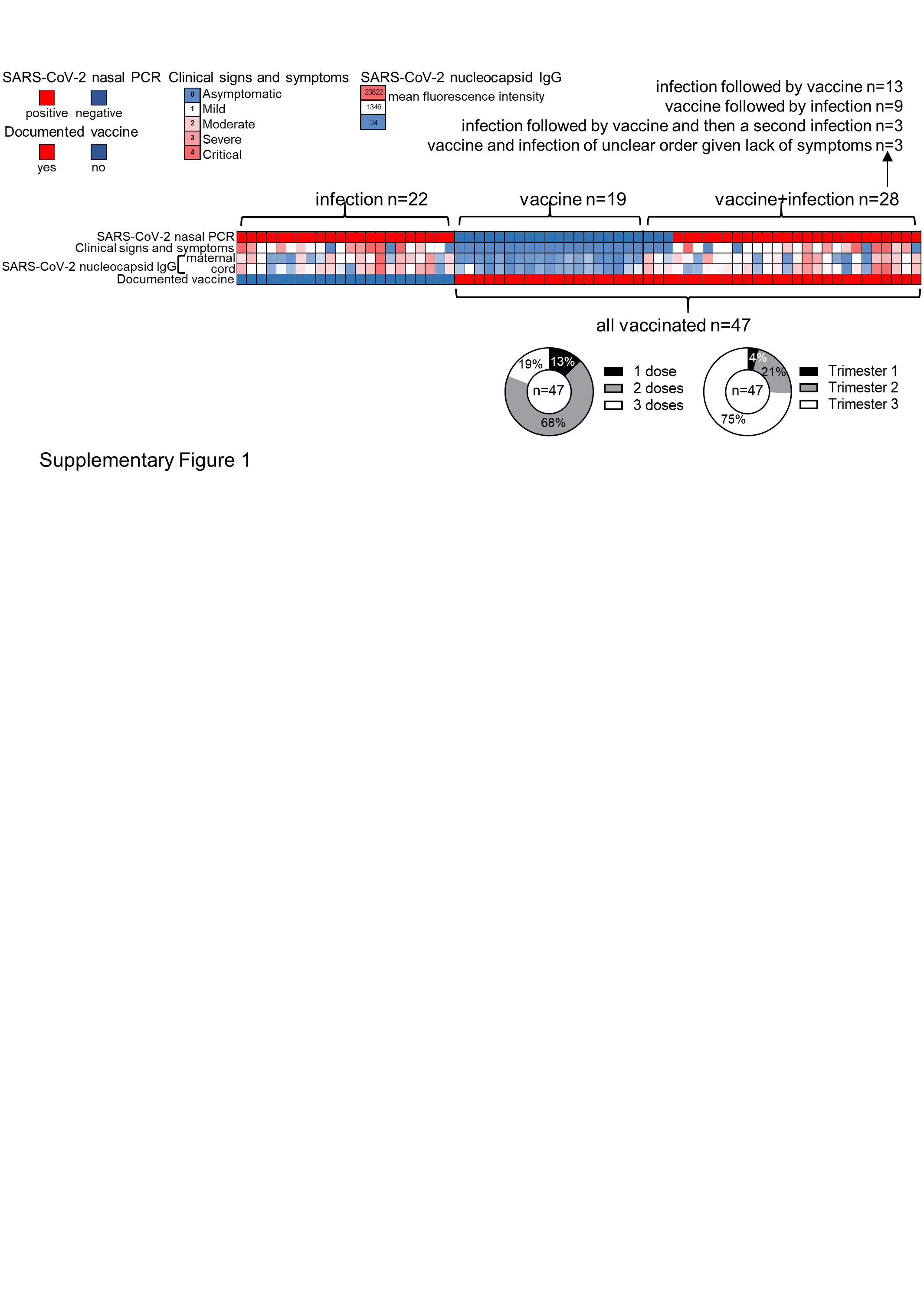

Supplement: jiad421_Supplementary_Data [file jiad421_supplementary_data.zip › Supplementary Figure 1.TIF]

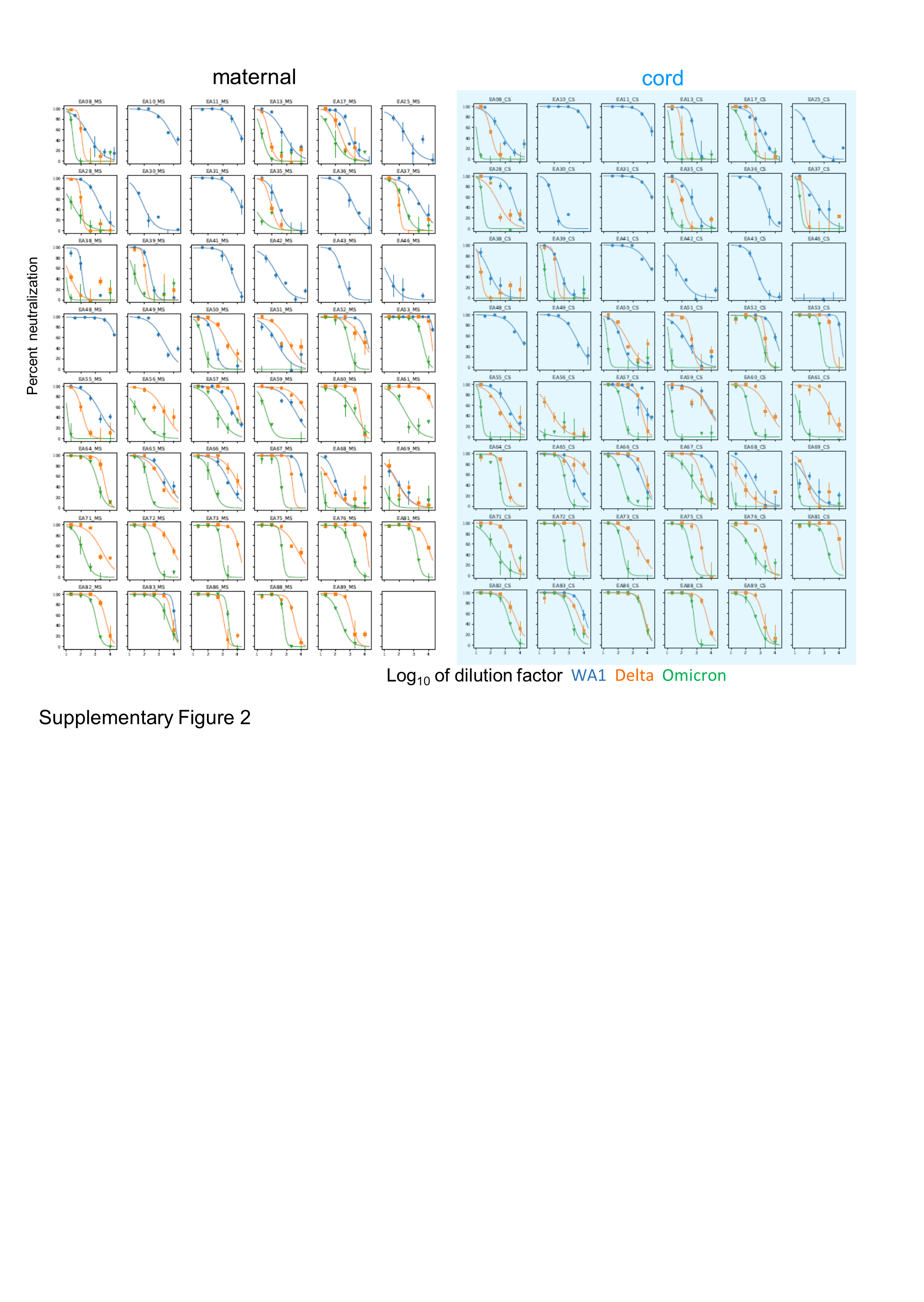

Supplement: jiad421_Supplementary_Data [file jiad421_supplementary_data.zip › Supplementary Figure 2.TIF]

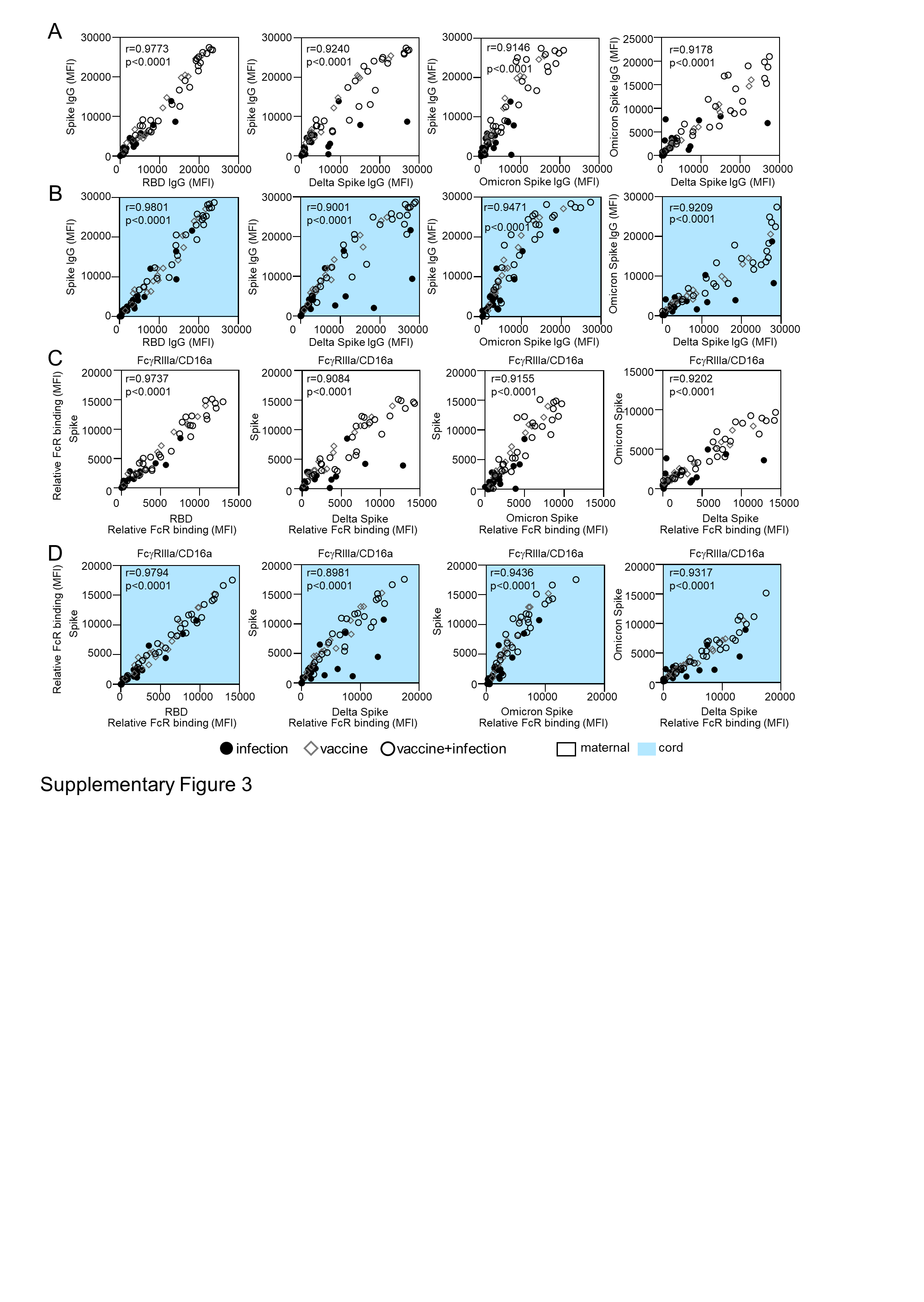

Supplement: jiad421_Supplementary_Data [file jiad421_supplementary_data.zip › Supplementary Figure 3.TIF]

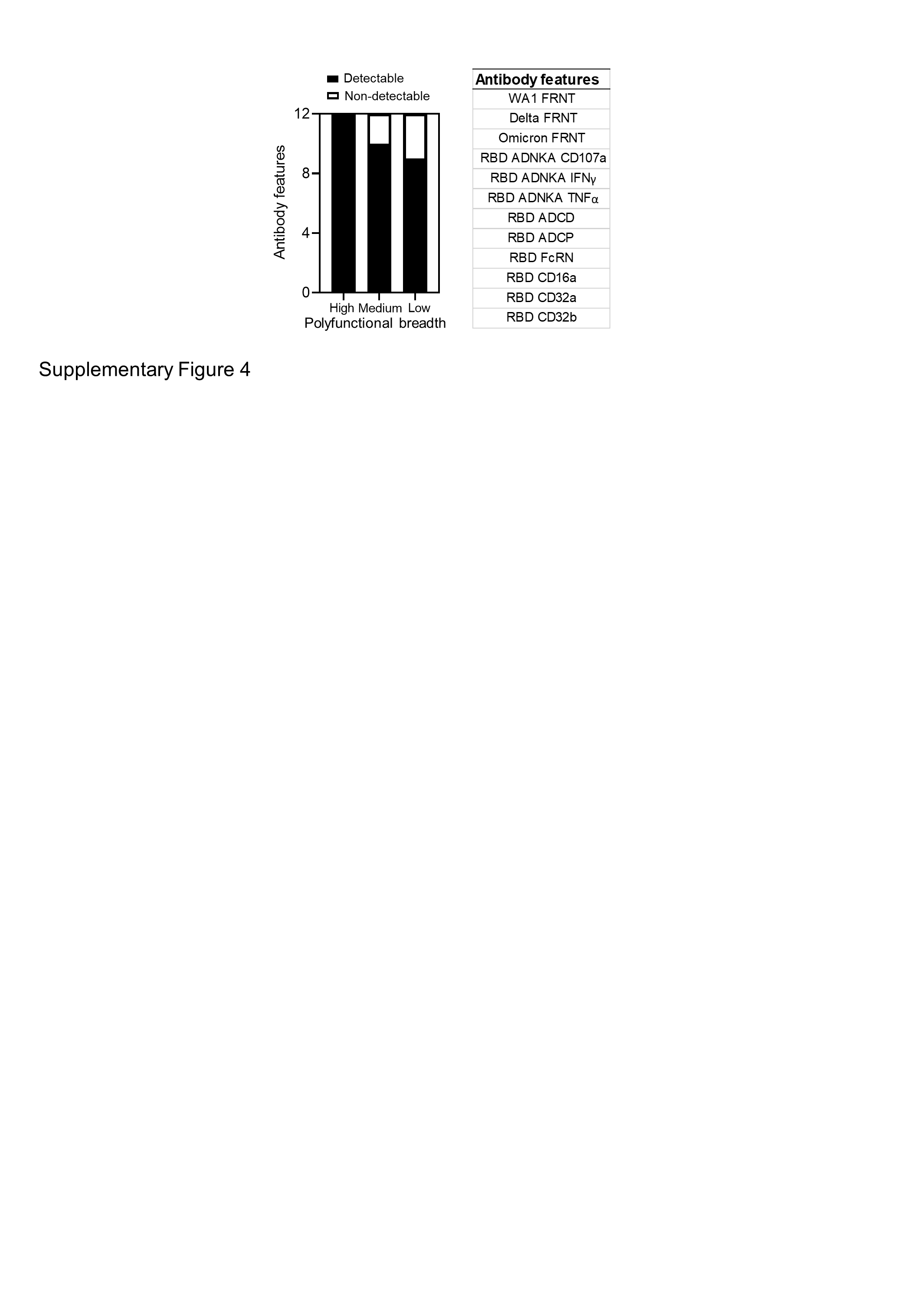

Supplement: jiad421_Supplementary_Data [file jiad421_supplementary_data.zip › Supplementary Figure 4.TIF]

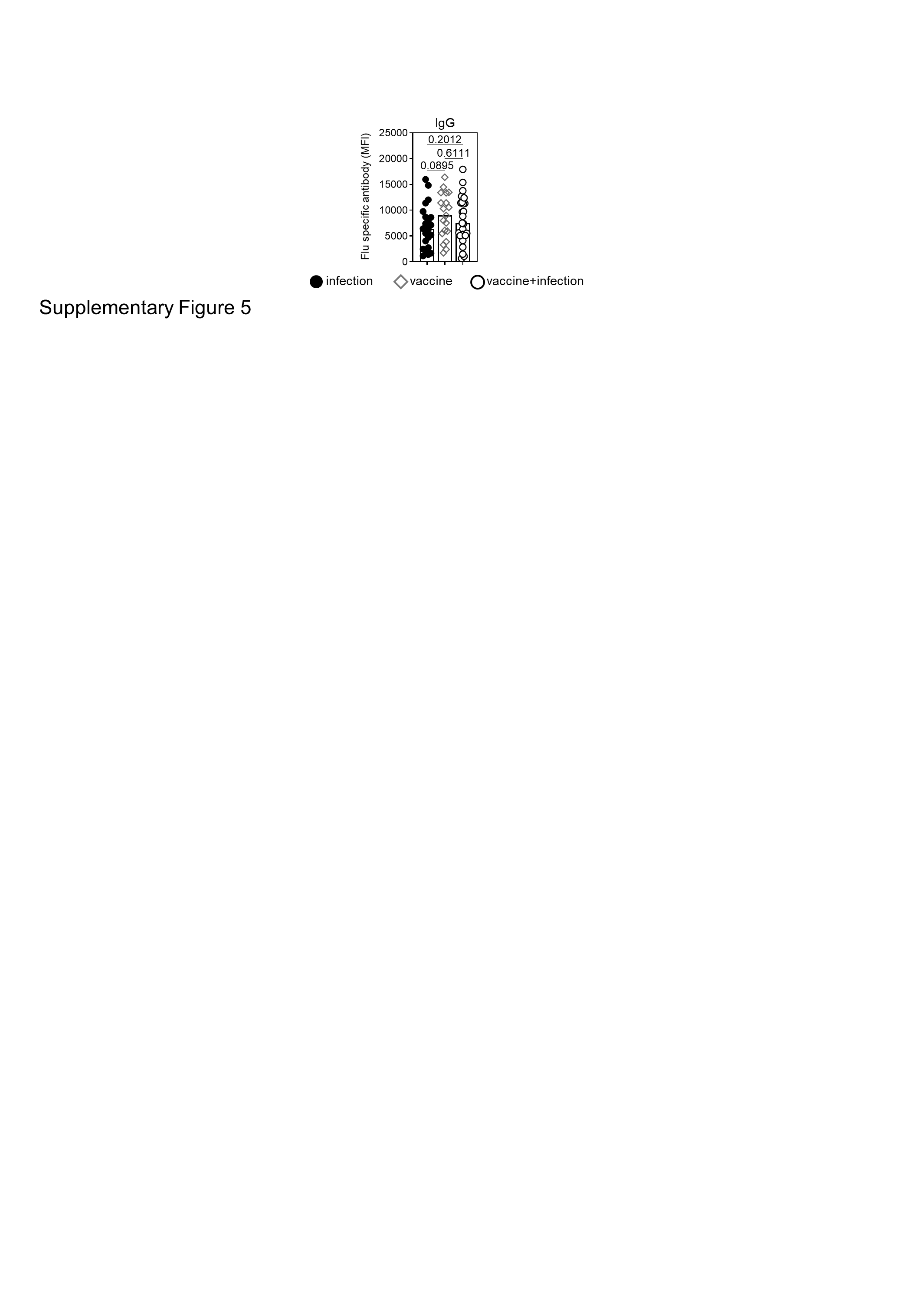

Supplement: jiad421_Supplementary_Data [file jiad421_supplementary_data.zip › Supplementary Figure 5.TIF]

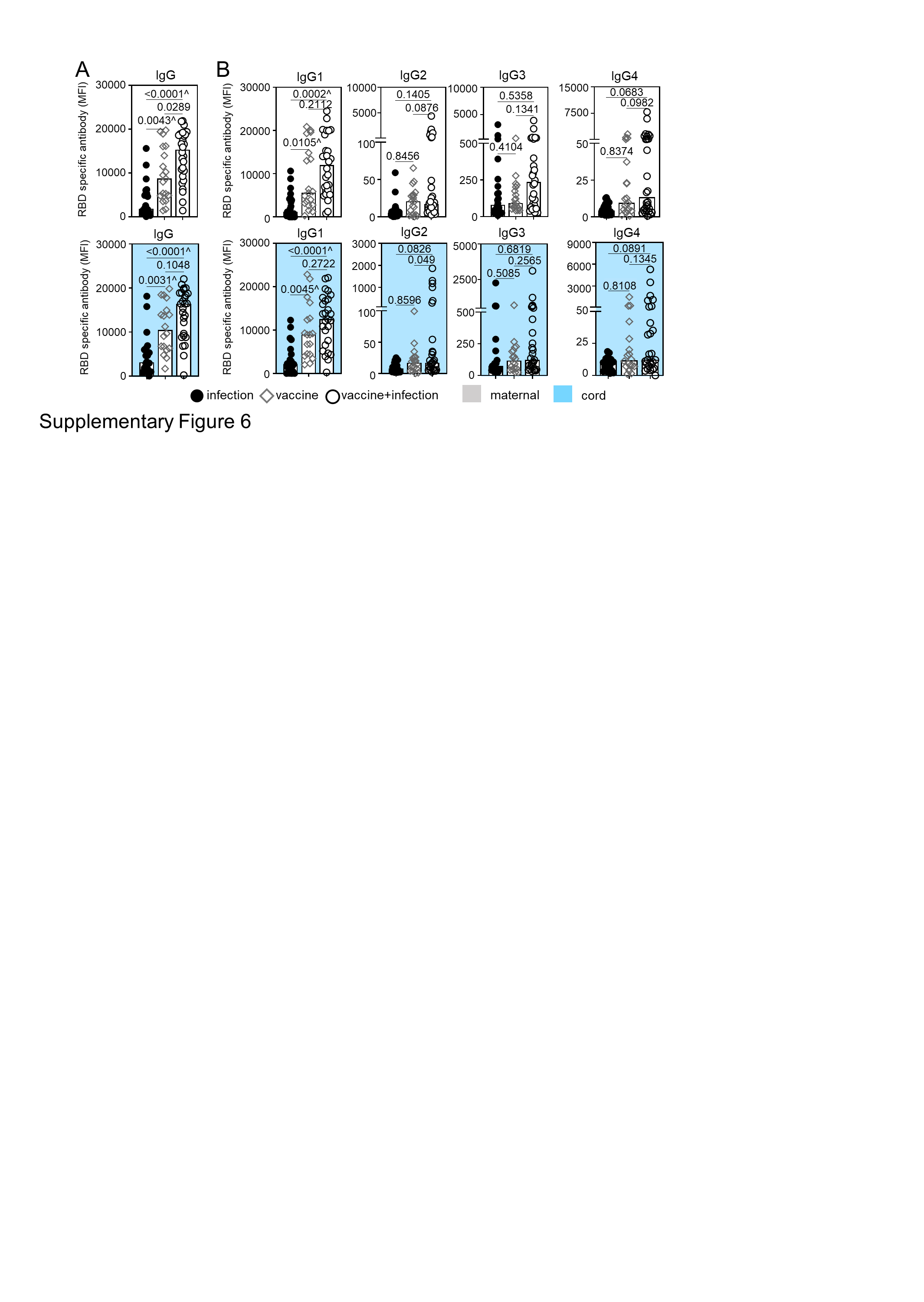

Supplement: jiad421_Supplementary_Data [file jiad421_supplementary_data.zip › Supplementary Figure 6.TIF]

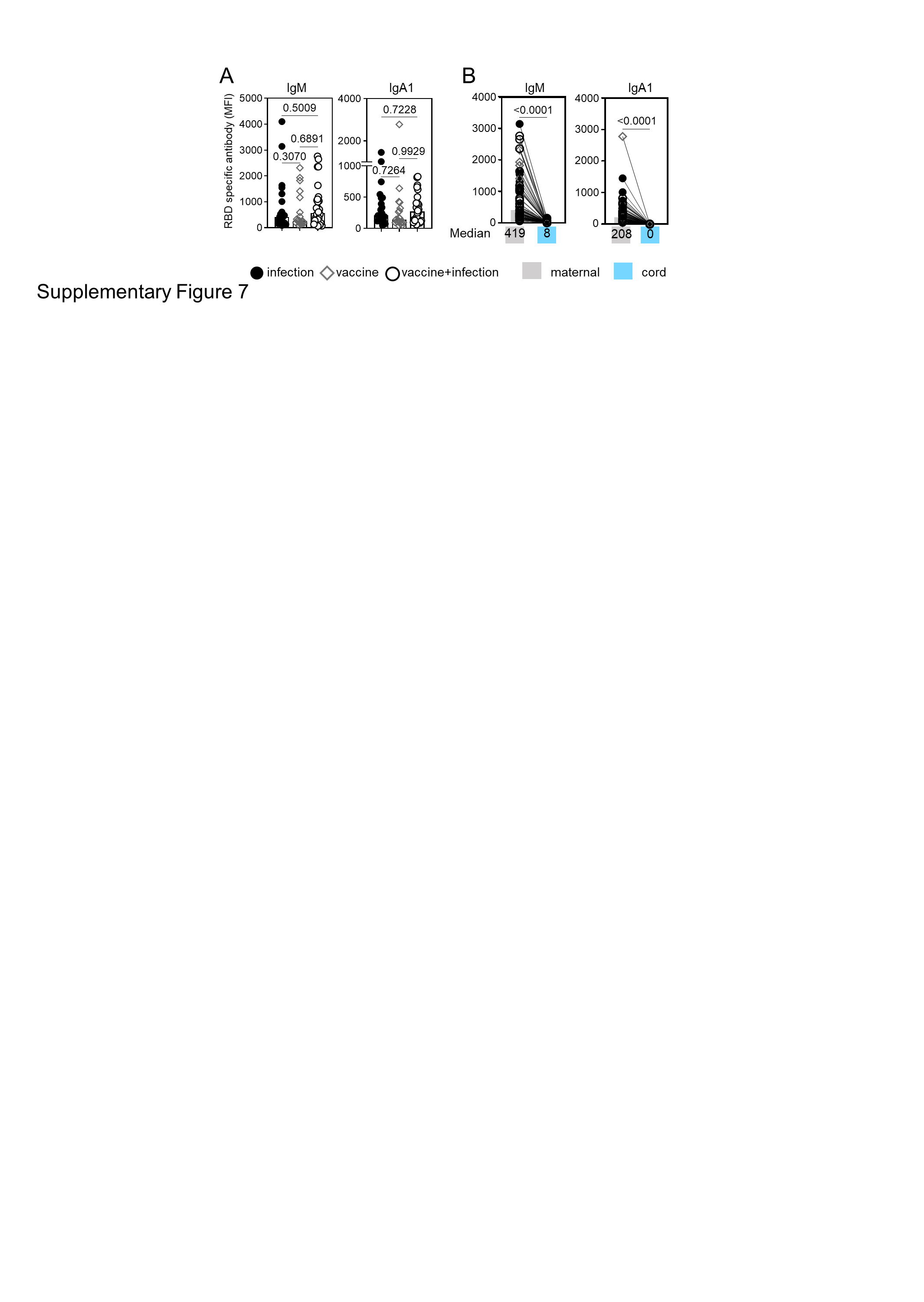

Supplement: jiad421_Supplementary_Data [file jiad421_supplementary_data.zip › Supplementary Figure 7.TIF]

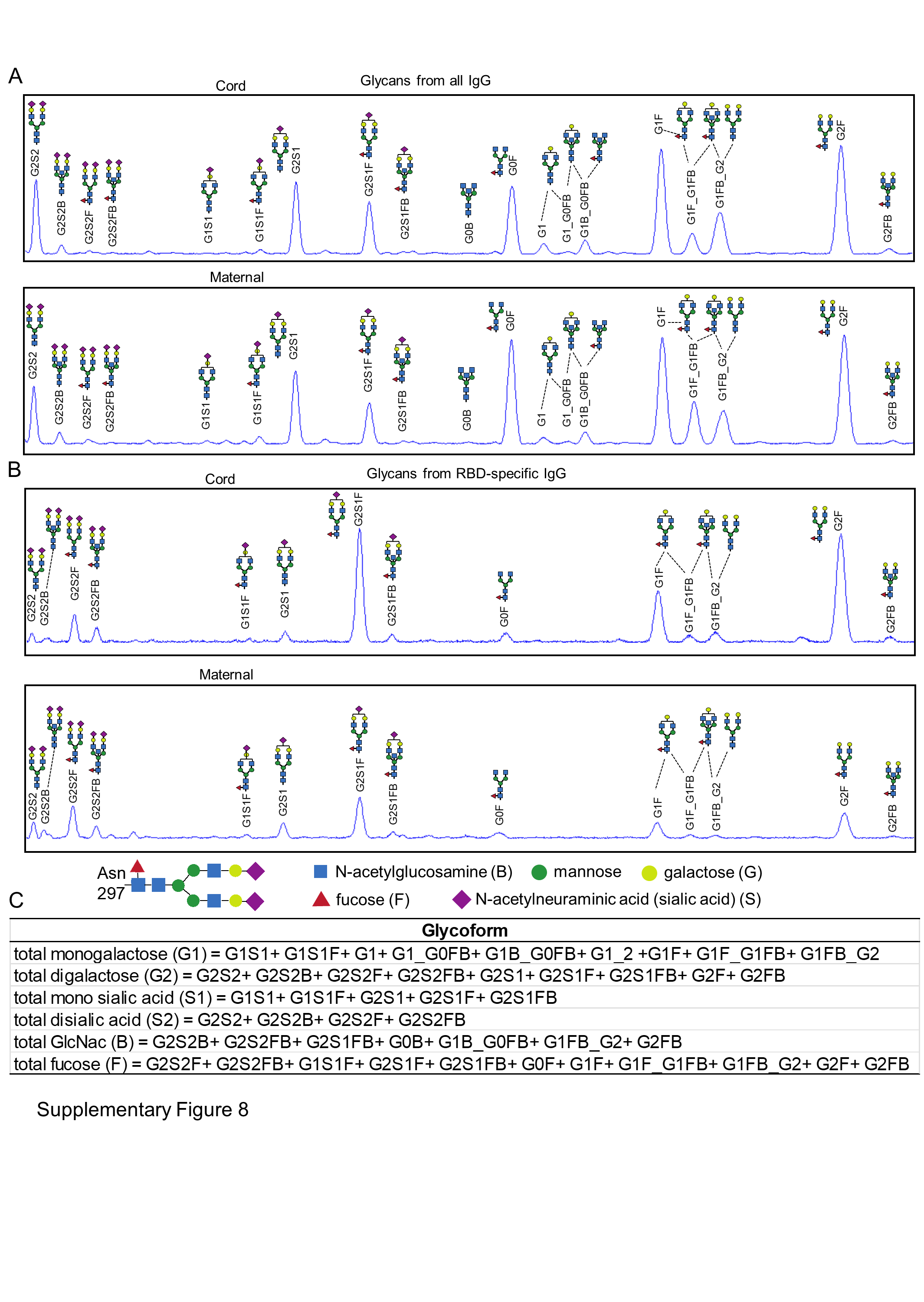

Supplement: jiad421_Supplementary_Data [file jiad421_supplementary_data.zip › Supplementary Figure 8.TIF]

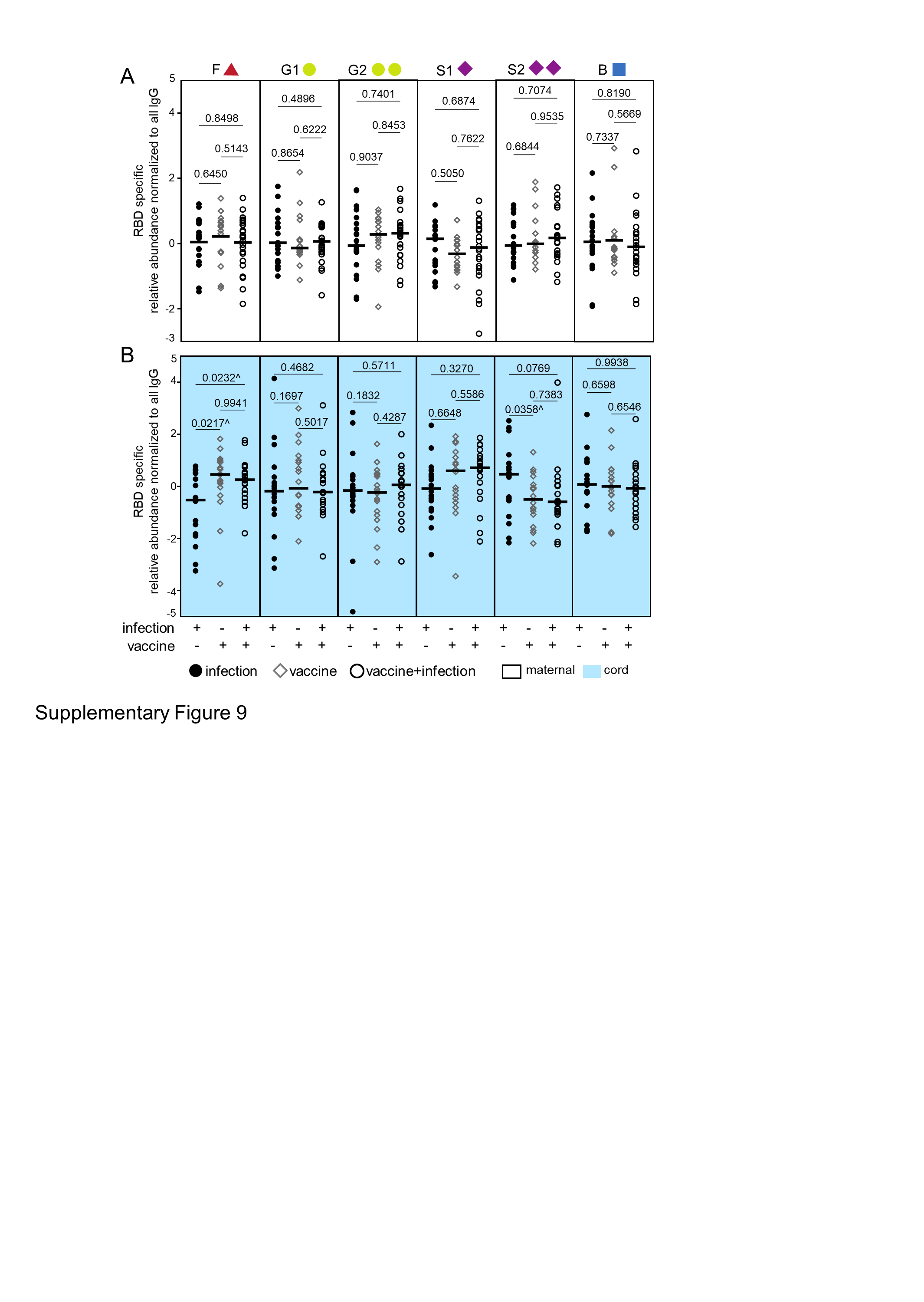

Supplement: jiad421_Supplementary_Data [file jiad421_supplementary_data.zip › Supplementary Figure 9.TIF]
